# Zeolitic Microporous Materials and Their Applications

**DOI:** 10.3390/molecules26030730

**Published:** 2021-01-31

**Authors:** Susana Valencia

**Affiliations:** Instituto de Tecnología Química (UPV-CSIC), Universitat Politècnica de València, Consejo Superior de Investigaciones Científicas, Av. de los Naranjos s/n, 46022 Valencia, Spain; svalenci@itq.upv.es

Research in the field of zeolites is a very active and relevant area, since these materials are still widely used as catalysts and adsorbents in many industrial applications, despite the appearance of other fascinating microporous materials with excellent properties. Every year several new zeolitic structures are reported, ranging from the 20 different zeolites known in the 1970s to the more than 250 reported so far [1]. This offers the possibility to select the appropriate zeolitic material that best fits the requirements of a particular process. Owing to the properties of zeolites, such as uniform porosity, thermal and hydrothermal stability, robustness, and the possibility of generating acidic or other active sites, these materials are applied in different areas. These include the use as selective catalysts in refining, petrochemical, and fine chemistry industries, also in the upgrading of new raw materials, and in adsorption and separation processes, among others.

This Special Issue of Molecules entitled “Zeolitic Microporous Materials and Their Applications” aims to collect original contributions on various topics related to zeolite materials, covering aspects ranging from the synthesis of zeolites, their characterization, and application in diverse fields. In particular, research papers on the use of natural zeolites, the synthesis of new zeolitic materials or the improvement of the properties of existing ones, their use as catalysts or adsorbents, and the structural and active sites characterization are reported in this Special Issue.

Qiu et al. [2] present a new zeolitic material, named SYSU-6, consisting of a 2-dimensional aluminophosphate zeolite layer precursor. The structure has been elucidated by single crystal X-ray diffraction and is composed of a new layer framework containing 4,12-ring linked by alternating AlO_4_ and PO_4_ tetrahedra. This new material has the potential of being the precursor of a new tridimensional zeolitic framework if the condensation of the layers is successfully achieved.

Articles describing the use of naturally occurring zeolites in different applications are reported by Presa et al. [3] and Kulikova et al. [4] First, natural mordenite zeolite from Spanish deposits is presented as a potentially useful additive for pozzolanic cements, as deduced from the good results of characterization data and tests carried out by the authors. They conclude that the characteristics are within the range of European requirements for commercial concretes and present the benefit of the low cost of the material [3]. Secondly, the use of natural zeolite minerals based on clinoptilolite-heulandite series from Ukrainian deposits are studied for managing radioactive wastes through their incorporation with magnesium potassium phosphates for achieving the solidification of the wastes. The authors conclude that the addition of zeolite increases the mechanical strength, as well as the resistance to radionuclide leaching of the final material, resulting in a useful alternative [4].

Several interesting studies about synthesis of zeolites with different topologies, structures and properties are also reported in this Special Issue [5,6,7,8]. The synthesis of MWW-type materials with intermediate physicochemical properties is investigated by Schwanke et al. [5] via the combination of hard- and soft-templating approaches in order to control the porosity of the materials. In this manner, it is possible to obtain MWW zeolites with pore systems comprising different porosities either in the interparticle and interlamellar ranges, which may potentially be useful as catalysts in reactions involving bulky reactants, and in the encapsulation of nanoparticles or biomolecules. A similar layered zeolitic material (Ferrierite) is the subject of the work carried out by Xu et al. [6] The authors show a simple synthesis strategy to control the thickness of the nanosheets based on the variation of the amount of organic ammonium which is used simultaneously as structure directing agent and inhibitor of crystal growth. As a consequence, the thickness of the ferrierite nanosheets can be nicely adjusted from 6 to 200 nm.

Mlekodaj et al. [7] report an interesting work on the synthesis of the large pore zeolite Beta in the absence of alkali cations using an aluminosilicate precursor. The alkali-free synthesis of zeolite Beta has already been reported previously, but the procedure described here allows the synthesis yields to be maximized and a less organic amount is required. Interestingly, the different post-synthesis treatments applied permit the control of the acid properties of the zeolite. 

The synthesis of zeolites in a different conformation is reported by Palomino et al. [8] who describe the preparation of continuous layers of the highly hydrophobic pure silica analogue of zeolite LTA (ITQ-29) which is grown on the surface of alumina supports. These layers are formed by the intergrowth of ITQ-29 zeolite crystals that are stable and free of cracks after calcination and are potentially applicable as membranes for separation of molecules based on their polarity.

This Special Issue also contains articles dealing with the use of zeolites in different catalytic applications. In particular, Erofeev et al. [9] report the effect of modifying MFI zeolites with zinc oxide on their catalytic behaviour in the conversion of the propane–butane fraction into arenes. The incorporation of zinc oxide produces a significant increased yield of liquid products and aromatic hydrocarbons which are even higher when the activation of the catalysts is done by low temperature plasma.

Agote-Arán et al. [10] study the potential use of the small pore zeolite SSZ-13 as a catalyst in the methane dehydroaromatisation reaction. The authors have investigated the structure of the Mo/H-SSZ-13 catalyst by operando X-ray absorption spectroscopy, in situ synchrotron powder diffraction, and electron microscopy. The results are compared with the medium pore zeolite ZSM-5 showing a lower catalytic performance of SSZ-13 attributed to the difficulties for attaining good dispersion of the Mo species inside the small pores of the zeolite. The authors conclude that further research is needed for optimizing the preparation of these catalysts based on small pore zeolites to evaluate their catalytic behaviour in this reaction.

The use of zeolitic materials in adsorption and separation processes either in gas and liquid phase has been also treated in this Special Issue. Sarti et al. [11] have studied the use of high silica commercial zeolites (Faujasite and Beta) in the adsorption of pharmaceuticals present in water. The results evidenced that the selected materials were efficient adsorbents towards all investigated molecules, as deduced from their high saturation capacities and adsorption constants in diluted aqueous solutions containing the pharmaceuticals. The zeolites have also shown good performance as pre-concentration media in dispersive-solid phase extraction experiments, leading to high recoveries.

Liu et al. [12] focus their attention on the magnetic solid-phase extraction of pesticides from water using composites formed by deep eutectic solvents coated on the surface of magnetic Zeolitic Imidazolate Framework-8 (ZIF-8). This is not exactly a zeolite material but a metal organic framework and the composites described here exhibit very interesting properties, such as superparamagnetism, large specific surface areas and porosity, allowing the adsorption of pyrethroids from water.

In the contribution from Narang et al. [13], the use of commercial low silica zeolites NaX (FAU) and CaA (LTA) as CO_2_ adsorbents for biogas upgrading is reported. The zeolite powders were structured by optimizing a freeze granulation process to form hierarchically porous granules able to perform the separation of CO_2_ from CH_4_. The materials have been characterized, the CO_2_ and CH_4_ isotherms have been measured, as well as breakthrough experiments. The NaX zeolite-based material exhibits higher CO_2_ uptake and selectivity than CaA and the shape of the adsorption–desorption cyclic breakthrough curves and mass transfer coefficient indicate that the granules offer lower mass transfer resistance than commercially available NaX granules. This is due to the creation of extra macroporosity during the freeze granulation process.

Characterization of zeolite materials is extremely important to evaluate their properties and all the articles of this Special Issue include this topic, but contributions especially focussed on advanced characterization are also reported here. Pyra et al. [14] have evaluated the features of zeolite Beta by using thermogravimetric analysis coupled with FTIR operando experiments in the cracking of low-density polyethylene (LDPE). The authors have studied zeolites Beta with different textural properties and have been able to evaluate the impact of textural and acidic parameters on the catalytic behaviour separately. It is mainly concluded that the presence of strong Si(OH)Al acid sites in the microporosity of the zeolite free of extraframework species favours the reduction of the maximum decomposition temperature of the polymer, that is, the activity in the catalytic cracking of LDPE is controlled by the intrinsic acidic character of the zeolite.

Finally, an interesting review about the characterization of the acidity of zeolites containing hierarchical porosity prepared by different approaches is reported by Gackowsky et al. [15] Hierarchical zeolites have attracted much attention in the last years since the presence of mesoporosity, besides the intrinsic microporosity of zeolites, enhances the accessibility of the reactants to the active sites in a catalytic reaction. Mesopore formation in zeolites can be attained by various methodologies, such as dealumination, desilication or by different templating methods using carbon particles, surfactants and other species, among others.

In particular, this review is focussed on hierarchical zeolites Y prepared by different procedures and places a special emphasis on the characterization of the acid properties. The authors first summarize the experimental techniques which are typically employed for acidity studies, as infrared (IR) and nuclear magnetic resonance (NMR) spectroscopies, microcalorimetry and thermoprogrammed desorption (TPD) of probe molecules. Later, the properties of desilicated zeolites Y are reviewed, paying attention to the acidity of the parent zeolites and after desilication, including the presence of extremely high acid strength sites, as well as to the Lewis acid sites. The characterization of hierarchical zeolites Y prepared by surfactant templating procedures is also revised, either for the materials prepared incorporating the surfactant during the synthesis of zeolite Y or in post-synthesis treatments. The review ends with another methodology for obtaining hierarchical zeolites based on the assembly of small zeolitic grains, which was also the topic of one of the contributions mentioned above [13]. In this case, the porosity is generated by modification of granules that contain a mixture of the zeolite with a binder, avoiding the use of expensive surfactants. The hierarchical zeolites discussed in this high-quality review show good behaviour as catalysts in isomerization, cracking and other catalytic reactions [15].

In summary, this Special Issue aims to provide an overview of the current state of the field of zeolite materials from different perspectives. All the authors who have contributed their excellent articles to this issue are very gratefully recognized as well as the reviewers who have done a highly valuable work. Last but not least, special thanks are devoted to the editorial team for their continuous help and support in the preparation of this Special Issue. Hopefully it will be attractive and inspiring to the Readers of Molecules.
